# Determinants of university students’ COVID-19 vaccination intentions and behavior

**DOI:** 10.1038/s41598-022-23044-9

**Published:** 2022-10-27

**Authors:** Markus Schäfer, Birgit Stark, Antonia M. Werner, Lina M. Mülder, Sebastian Heller, Jennifer L. Reichel, Lisa Schwab, Thomas Rigotti, Manfred E. Beutel, Perikles Simon, Stephan Letzel, Pavel Dietz

**Affiliations:** 1 Department of Communication, Johannes Gutenberg University, Mainz, Germany; 2 Department of Psychosomatic Medicine and Psychotherapy, University Medical Center of the Johannes Gutenberg University, Mainz, Germany; 3 Department of Work, Organizational, and Business Psychology, Institute for Psychology, Johannes Gutenberg University, Mainz, Germany; 4 Institute of Occupational, Social, and Environmental Medicine, University Medical Center of the Johannes Gutenberg University, Mainz, Germany; 5 Department of Sports Medicine, Rehabilitation and Disease Prevention, Institute of Sport Science, Johannes Gutenberg University, Mainz, Germany; 6 Leibniz Institute for Resilience Research, Mainz, Germany

**Keywords:** Diseases, Psychology, Human behaviour, Disease prevention, Public health

## Abstract

Vaccination hesitancy has been a major challenge for health authorities and societies during the COVID-19 pandemic. The general determinants of vaccination intentions and behavior include sociodemographic and health-related, psychological, and communication-related factors, with varying relevance for different types of vaccinations, countries, and target groups. The predictors of university students’ COVID-19 vaccination behavior have not been sufficiently studied. Using a German university as an example and based on an online survey of 1398 university students, we investigated factors related to (a) the likelihood of vaccination against COVID-19 and (b) vaccination intention among those who had not been vaccinated by summer of 2021. Psychological factors showed high relevance. Field of study, trust in, and use of certain media and information channels were found to be important determinants of students’ vaccination decision. Our findings can help better understand university students’ vaccination behavior to develop and implement tailored strategies and campaigns.

## Introduction

Vaccinations are an important achievement of modern medicine, preventing several million deaths every year, including those from the ongoing COVID-19 pandemic^1,2^. Therefore, the World Health Organization (WHO) considers rapid access to effective vaccines as one of the most important aspects of global health and vaccination hesitancy as one of the leading global health threats^2,3^. Vaccination hesitancy is defined as delayed acceptance or rejection of a vaccination despite its availability and efficacy^4,5^. In terms of COVID-19, the resulting vaccination gaps have repeatedly posed great challenges to health authorities and societies around the world^6^. Therefore, the identification and investigation of influencing factors that can shape vaccination decision are crucial not only from a scientific perspective but also from a public health management perspective.

Whether a person is willing to get vaccinated (against a certain disease) is not a stable trait but a context-specific state on a continuum. Therefore, actual vaccination behavior can vary depending on, for example, time and place, the type of vaccination and vaccine, the legal framework, individual access, time resources, and whether or not an appointment is remembered^4,5,7,8^. Potential factors influencing vaccination intentions and behavior include *sociodemographic factors*, such as age, gender, education, and social background^9,10^; *health-related factors*, such as the presence or absence of chronic diseases^11–13^ or the health literacy of a person^14,15^; *psychological factors*, such as trust in vaccination safety or the perception of risks and barriers^4,9,16–18^; and *communication-related factors*, such as (general and topic-related) trust in and use of mass media and other information channels^19–27^.

In the past, these factors showed varying relevance for different types of vaccinations, with differences also observed between countries and regions^4,5,7,8,16,28^. For COVID-19 vaccination, the evidence is still inconsistent, and many questions remain open. Most international studies have tended to find that women are more vaccination hesitant than men^6,18,29,30^. Different or no clear tendencies were found for age and formal education^6,18,29–31^. In some studies, the presence of certain chronic diseases was associated with an increased intention to get vaccinated, but this was not reported for others^11,12^. A comparatively clear trend emerged that, as with other vaccinations, confidence in the safety and effectiveness of vaccination^29,30,32^ and the perception of COVID-19 as a risk^6,32^ tended to be positively associated with the intention to get vaccinated. Acceptance of and recent experience with other vaccinations were associated with a higher intention to get vaccinated against COVID-19 in some cases^6^. Furthermore, some findings suggested that contact with misinformation was negatively associated with vaccination intention^27^ and that those who were hesitant tended to report lower general media use, lower trust in and lower use of information from traditional mass media, authorities, and representatives of the health system, and higher trust in information from social media and alternative news sources^17,18,22^.

During the COVID-19 pandemic, university students, as an active, young, and mobile subpopulation, have repeatedly come into the focus of pandemic management^33,34^. Due to their age structure, their societal multiplier function, and their living and working conditions at colleges and universities, students are a particularly important target group for health prevention and promotion in general^35^. Specifically, they are for COVID-19 vaccination campaigns, given that international findings have shown that, for different countries, relevant proportions of the student subpopulation are skeptical or hesitant about whether they should get vaccinated against COVID-19^32,36–39^. However, it must also be taken into account in this context that, at least for as long as COVID-19 vaccines were in short supply, university students in general in many countries were not considered among the prioritized groups in vaccine distribution.

Preliminary studies on this target group have suggested that at least some of the general determinants of vaccination intentions and behaviors may also be relevant among university students^32,36–40^. A study on students from universities in the Netherlands, Belgium, and Portugal revealed that confidence in the safety and effectiveness of vaccination, the perception of the disease as a risk, and the perception of a responsibility to protect others by getting vaccinated were positively associated with the intention to get vaccinated, whereas the perception of barriers and the weighing of benefits and risks of vaccination were negatively associated with the intention to get vaccinated^32^. For Czech university students, trust in healthcare providers and the use of social media were identified to be positively and negatively related to the intention to vaccinate against COVID-19, respectively^38^. In a US sample, a higher/lower trust in official information sources and news media regarding COVID-19 vaccines was associated with a higher/lower acceptance of vaccination, while no association was reported for trust in social media, family, and friends as news sources^37^. For different countries, confidence in the efficacy and safety of other vaccinations and an actual previous vaccination against other infectious diseases were reported to be negatively related to COVID-19-vaccination hesitancy^37,39^. According to a study from France, female students were more hesitant about vaccination than males^39^. Conversely, other studies conducted in different countries and with different student subpopulations showed no gender differences in vaccination intention^36–38,40^.

Although general findings are not entirely consistent^6,41^, it can be further hypothesized that the field of study can also be an important factor for students’ vaccination decision^36,39,40^. Due to their study-related proximity to the healthcare sector and the subject of infectious diseases and vaccinations, medical students may be more aware of the relevance of vaccination and probably more motivated to get vaccinated than students from other disciplines. Furthermore, due to possible practical activities in the health sector, medical students may also have better and earlier access to health professionals and COVID-19 vaccines than their fellow students^37^. Nevertheless, empirical findings are inconsistent: while French healthcare students showed a higher intention to vaccinate than students from other disciplines^39^, for university students in Italy, no differences between healthcare and non-healthcare students were reported^36^.

Taken together, the predictors of university students’ COVID-19 vaccination intentions and behavior need further attention. Previous international research has produced mixed findings, and there is a lack of studies dealing with the interaction of different indicators with *vaccination behavior* (i.e., the behavior of having (not) taken vaccination against COVID-19 at least once with an approved vaccine), as reported *intentions* do not always translate into vaccination uptake^42^. However, a better understanding is important to achieve a broader immunization of the target group by developing and implementing appropriate tailored measures. This immunization among students has emerged as an important key to a functioning and healthy university life during the past months, given that without it, maintaining regular attendance during the COVID-19 pandemic repeatedly proved difficult at many universities, with far-reaching consequences for the mental well-being of students^43–45^. Using the example of a large German university, the present study i) investigates how *sociodemographic and study-related, health-related, psychological,* and *communication-related* factors were associated with (a) the likelihood of vaccination against COVID-19 among university students and (b) the vaccination intention among those who had not been vaccinated in summer of 2021. Our results may help to better understand the determinants of university students’ vaccination behavior to develop and implement tailored strategies and campaigns for the target group to reduce vaccination hesitancy among university students.

## Methods

### Study design, participants, and procedure

An online survey was conducted between June 21 and August 15, 2021 at the Johannes Gutenberg University Mainz, a large German comprehensive university (about 31,500 students), with a full range of disciplines and subjects, located in the German mid-sized city Mainz (about 210,000 inhabitants). Using the university’s central mailing list, all students of the university were invited to a cross-sectional online health survey in the summer term 2021 as part of an ongoing health promotion project among students ("Healthy Campus Mainz"). To reduce a possible selection bias, we chose a mixed incentive strategy: A lottery of gift cards for a major international online shop and a local online food provider (total value: 500 €) served as the monetary incentive, and the announcement of a donation (1.000 €) to a local pediatric cancer center upon reaching a certain number of participants served as the non-monetary incentive. Reminder emails were sent twice. At the beginning of the online questionnaire, we explained the background and purpose of the study and informed the participants that participation would be anonymous and voluntary. Informed consent from all participants was obtained.

### Vaccination situation in Germany at the time of study

The academic year at most German universities is divided in two terms: a winter semester, which usually starts mid-October, and a summer semester, which starts mid-April. At the time of our survey, university classes were held exclusively online, with only a few exceptions possible (e.g., laboratory practical training). At this time, four vaccines against COVID-19 were licensed in the European Union: mRNA vaccines from BioNtech/Pfizer and Moderna and vector vaccines from AstraZeneca and Johnson & Johnson^46^. In Germany, people were initially vaccinated in specially created vaccination centers and by mobile vaccination teams. Eventually, doctors and hospitals were included in the vaccination campaign^44^. The German campaign officially began on December 27, 2020^48^. A staged procedure was used for vaccination prioritization because of the scarce vaccine quantities^49^ and was adapted several times. The oldest citizens aged 80 and above were prioritized. Priority was also given to residents of nursing homes, staff of medical facilities, and people suffering from certain chronic diseases^49,50^. Later, teachers, retail workers, members of the critical infrastructure, and generally people with precarious working and/or living conditions and/or an increased risk of exposure were also included in the prioritization^50^. On June 7, 2021, the German federal government finally ended vaccination prioritization^51^, making it possible for everyone over 18 years to get COVID-19 vaccination. However, German vaccine stocks remained in short supply for several more weeks, and vaccination appointments were not always immediately available.

### Measures

#### Dependent variables

To assess their COVID-19 vaccination status, we asked a single question: “Have you already been vaccinated against COVID-19?” The respondents specified whether they had not been vaccinated (“no”), had been vaccinated once (“yes, once”), or had been vaccinated twice (“yes, twice”) with a COVID-19 vaccine. For analysis, we merged the last two response categories into a single response category for vaccination uptake, resulting in a binary variable (0 = no vaccination, 1 = at least one vaccination). For students who indicated that they had not been vaccinated, we further asked for their COVID-19 vaccination intention (“How likely is it that you will get vaccinated if you are offered vaccination against COVID-19?”). The respondents specified their answers on a scale from 1 (“very unlikely”) to 11 (“very likely”).

#### Independent variables

##### Sociodemographic and study-related factors

The sociodemographic and study-related information collected from the questionnaire included *age* (recorded in years as a continuous variable), *gender* (male, female, diverse, open), and *field of study*. For the latter, we provided the respondents with a list of all available subjects at the university (e.g., art history, dentistry, economics, journalism, law, meterology, musical science, physics, philosophie, psychology, theology, etc.; “Please select your (main) field of study from the list below. If you are studying more than one subject, please indicate the field you consider to be the main focus of your studies.”). For analyses, field of study was prepared as a binary variable, with medicine (1) and other fields of study (0) as binary options.

##### Health-related factors

*General health status* was assessed on a scale from 0 to 10 with one question: “If you rate the best conceivable state of health with 10 points and the worst conceivable with 0 points, how many points would you award your current state of health?”. We further measured *general well-being* using the German version of the WHO-5 Well-Being Index (WHO-5)^52^, which asks respondents to rate how well each of five statements applies to them when considering the last 14 days (“I have felt cheerful and in good spirits,” “I have felt calm and relaxed,” “I have felt active and vigorous,” “I woke up feeling fresh and rested,” and “My daily life has been filled with things that interest me”). Each item is scored from 0 (none of the time) to 5 (all of the time). The *presence of a chronic disease or disability* was asked two yes/no questions: “Have you been diagnosed with a chronic disease?” and “Have you been diagnosed with a disability?”. For analysis, we combined both answers into a single binary item (presence of a chronic disease or disability (yes = 1/no = 0).

We assessed *health literacy* with one item for each of the four dimensions (ability to search, understand, evaluate, and apply health information) of the German health literacy scale^53^. The questions were as follows: “How easy/difficult is it to…” (a) “…find information about symptoms of illness that affect you?”, (b) “…understand what to do in a medical emergency?”, (c) “…judge when you should see a health professional for a check-up?”, and (d) “…make decisions that will improve your health?”. Answers were recorded on a four-point scale from 0 to 3 (3 = “very easy,” 2 = “fairly easy,” 1 = “fairly difficult,” 0 = “very difficult”), resulting in an overall summary index for health literacy from 0 to 12. A score of 12 indicates high health literacy, while a score of 0 suggests poor health literacy. Good health literacy is assumed in the range of 8 to 12, while a score of 4 and below is considered to be weak health literacy. General interest in common vaccinations was assessed by the question, “How important is it to you to have adequate vaccination protection against common diseases (e.g., mumps, measles, rubella, tetanus)?”. The participants indicated their answers on a bipolar five-point scale from 1 (“not important at all”) to 5 (“very important”).

##### Psychological factors

We measured the psychological determinants of vaccination using the short scale of the 5C model^4^ adopted for COVID-19, which differentiates five dimensions of influence: confidence, complacency, constraints, collective responsibility, and calculation. “Confidence” is defined as the level of trust in the effectiveness and safety of immunization. “Complacency” refers to the individually perceived risk of disease, “constraints” to the individually perceived structural barriers in everyday life, and the motivation to overcome them. “Collective responsibility” is the motivation to help protect others by getting vaccinated, “calculation” the extent of conscious evaluation of the benefits and risks of vaccination. The five items were as follows: “I am completely confident that vaccination against COVID-19 is safe” (*confidence*); “Vaccination is unnecessary because COVID-19 is not a major threat” (*complacency*); “Everyday stress prevents me from getting vaccinated against COVID-19” (*constraints*); “When everyone is vaccinated against COVID-19, I don’t have to get vaccinated, too” (*collective responsibility*); and “When I think about getting vaccinated against COVID-19, I weigh the benefits and risks to make the best decision possible” (*calculation*). Each statement was rated on a seven-point scale (1 = “do not agree at all,” 7 = “fully agree”).

##### Communication-related factors

We assessed the general and COVID-19-specific trust in mass media and certain information channels, the intensity of general media use and COVID-19-related information seeking, and the use of certain information channels. *General media trust* and *topic-specific media trust* were measured with single questions (“In general terms, how much do you think the media in Germany can be trusted?”; “There is currently a lot of media coverage of the COVID-19 pandemic. How much can the media in Germany be trusted on this topic?”). For both items, the answers were graded on a five-point scale (1 = “not at all,” 5 = “completely”). On the same scale, general trust was recorded for *specific media outlets*, including their off- and online appearances (e.g., public and private broadcasting, quality press, tabloid media, alternative media, social media, messenger services, and video platforms). We further asked the participants to indicate, again on a five-point scale, their topic-related *trust in certain information sources* (e.g., German federal government, Robert Koch Institute (RKI; Germany’s national institute for disease control and prevention), WHO, medical staff, universities, scientists, political parties, politicians, and common people) regarding COVID-19 (“How trustworthy are the following sources with regard to the coronavirus topic?”; 1 = “not at all trustworthy,” 5 = “very trustworthy”).

*Intensity of general media use* (radio, TV, print media, and internet) and *COVID-19 information seeking* were recorded in days per week (0–7). The participants indicated whether they had used certain sources for information on COVID-19 in the preceding year (e.g., interpersonal sources: family members, friends, colleagues, health professionals, mass media sources, and social media) through the following question: “Where have you gathered information on the coronavirus topic during the past 12 months?”. Multiple answers were possible. Subsequently, the participants who indicated they had used online sources were asked whether they had used certain online sources (e.g., websites of health professionals, journalistic online news media, COVID-19 warning app, blogs, social media, and video platforms). Again, multiple answers were possible. For analyses, the non-use of a certain source was coded low (0), and the use of a certain source was coded high (1).

### Data analysis

Statistical analyses were conducted using the Statistical Package for the Social Sciences (IBM SPSS version 23). Descriptive statistics are presented as means (M) with standard deviations (SD) for continuous scaled variables and as numbers and percentages for non-continuous scaled variables.

For regression analyses, we used Cohen’s f^2^ as an additional standardized measure of effect size, which can take on values between zero and an indefinitely large number^54,55^. Values of 0.10 represent small, 0.25 medium, and 0.40 large effect sizes.

To predict students’ vaccination status by the summer term of 2021, we conducted a binary logistic regression. To predict vaccination intention among unvaccinated students, we conducted a multiple linear regression. A total of 1,438 students participated in the survey. Among these, 1398 indicated their vaccination status. The sample size varied between some independent variables because of the answering options, which also included the possibility of not providing information. For regression analyses, we decided to use listwise deletion. Thus, the final binary logistic regression model for vaccination status was based on a complete dataset of 1114 participants. The final linear regression model for the vaccination intention of unvaccinated students was based on 334 complete datasets.

We found no fundamental problems either regarding homoscedasticity, normality or linearity of relationship. To decide which variables to include in the regression models, we chose a mixed approach based on assumptions and background knowledge provided by the findings of previous research and empirical variable selection. Before binary logistic regression, preceding basic analyses using t-tests for the mean comparisons between vaccinated and unvaccinated students for continuous variables and Pearson’s chi-square test (χ^2^) for categorial variables were conducted for each independent variable. Only variables that showed significant differences between vaccinated and unvaccinated students (*p* < 0.05) in the basic comparisons were included in the main analysis. To decide which variables to include in the multiple linear regression, we conducted t-tests and ANOVA regarding the vaccination intention of unvaccinated students. We further calculated bivariate correlations between all metric variables and vaccination intention to see if they were statistically related. Again, only variables that showed significant differences or correlations in the basic analyses (*p* < 0.05) entered main analysis.

We provide a list of all items used for the present analyses and their specific questions and answering options, as well as an overview of the details of the basic analyses, in the supplementary material.

To avoid multicollinearity, we checked all selected independent variables for high correlations (r > 0.70). For the variables included in the binary logistic regression and the variables included in the multiple linear regression, this applied to the variables “trust in the federal government” and “trust in the state government” which were highly correlated (r = 0.76 / r = 0.80). We therefore decided to remove the second variable and to keep only “trust in the federal government” for main analyses.

The variance inflation factor (VIF), a measure of the degree of multicollinearity in a set of multiple regression variables, was calculated for all remaining variables entering main analysis. This value was less than 2 for most and less than 3.5 for all variables, indicating low to moderate multicollinearity, but no fundamental problems.

### Ethics approval

The manuscript has been read and approved by all named authors. Approval to perform the study was given by the ethical committee of the Institute of Psychology of Johannes Gutenberg University (JGU) Mainz (application-number: 2021-JGU-psychEK-S017). The study was performed in accordance with the World Medical Association (WMA) Declaration of Helsinki on the ethical principles for medical research involving human subjects and the Ethical Principles and Guidelines for the Protection of Human Subjects of Research by the American Psychological Association (APA). Our study did not involve an experimental design.

## Results

### Main analysis I: Vaccination status (all students)

In the final sample (n = 1114), younger and female students were overrepresented in the participants in terms of the distribution of the sociodemographic characteristics at the university and in the student body in Germany as a whole (Table 1). The latter reflects a tendency observed in other recent student health surveys at European universities^36,38,39^. More than two-thirds (69.9%) of the students in the final sample had already received at least one dose of vaccination against COVID-19 in the summer semester of 2021, while about one-third (30.1%) had not been vaccinated against COVID-19.

**Table 1:** Distribution of age and gender of all participants in the final sample, among students at the corresponding university, and in the student body in Germany.

	Sample (N = 1,114)	University (N = 31,194)	Germany (N = 2.9 Mio.)
Age	M = 23.5	M = 24.7	M = 23.4
Gender			
male	24.3%	41.0%	50.2%
female	73.4%	59.0%	49.8%
diverse	0.9%	–	–
open	1.3%	–	–

The binary logistic regression model was statistically significant (χ^2^(44) = 276.33, *p* < 0.001, n = 1,114) and explained 31.1% of variance in university students’ vaccination status (Nagelkerke’s R^2^ = 0.311), resulting in a large Cohen’s f effect size of f^2^ = 0.45. The overall percentage of accuracy in classification was 71.8%, with a sensitivity of 73.7% and a specificity of 67.5%.

Of the variables entered into the regression model after basic analyses, nine contributed significantly to predicting COVID-19 vaccination behavior (Table 2). The relative likelihood for university students to be vaccinated by the summer term of 2021 was 2.13-fold when they were enrolled in medical school. By contrast, age, health literacy, and the relevance attributed to getting common vaccinations did not have a significant influence on the likelihood of being vaccinated. Vaccination was more likely in those who had greater confidence in the safety and effectiveness of COVID-19 vaccination. It was less likely the more the students weighed the pros and cons of a vaccination (calculation) and the more they perceived subjective barriers to vaccination (constraints). There was no significant association between the likelihood of vaccination and individual risk perception (complacency), the perception of a collective responsibility to get vaccinated, general and topic-specific media trust, or trust in specific traditional journalistic news sources, such as private and public broadcasting and national quality newspapers, including their respective online outlets. Conversely, stronger trust in social media as an information channel was associated with a higher and stronger trust in alternative news media and blogs with a lower relative likelihood for a COVID-19 vaccination. Topic-specific trust in the German federal government, political parties, and individual politicians, the WHO, the RKI, the national commission on vaccination, the national board of ethics, public health offices and hospitals were not significantly associated with the likeliness to be vaccinated in our model. Neither did the intensity of the general use of television, radio, print media, and online media. By contrast, topic-related trust in churches was positively associated with the likelihood of COVID-19 vaccination.

**Table 2:** Associations of sociodemographic, study-related, psychological, health-related, and communication-related factors with the likelihood of a vaccination against COVID-19 among university students in the summer of 2021.

	OR (95% CI)	Wald	*p*
**Sociodemographic and study-related factors**			
Age	1.036 (0.994–1.080)	2.82	.093
Field of study (with medicine coded high)	2.125 (1.230–3.672)	7.30**	.007
**Health-related factors**			
Health literacy	0.989 (0.918–1.066)	0.08	.779
Global interest in common vaccinations	1.017 (0.824–1.256)	0.03	.873
**Psychological factors**			
Confidence	1.424 (1.239–1.638)	24.65***	< .001
Complacency	0.966 (0.784–1.190)	0.11	.744
Constraints	0.618 (0.521–0.733)	30.65***	< .001
Calculation	0.878 (0.805–0.957)	8.77**	.003
Collective Responsibility	0.869 (0.747–1.011)	3.29	.070
**Communication-related factors**			
**Media Trust**			
General media trust	0.912 (0.719–1.157)	0.58	.448
Topic-specific media trust	0.845 (0.661–1.079)	1.83	.176
Public broadcasting (including online outlets)	0.962 (0.750–1.235)	0.09	.761
Private broadcasting (including online outlets)	0.999 (0.830–1.203)	0.00	.995
National quality press (including online outlets)	0.900 (0.717–1.129)	0.83	.363
Social media	1.355 (1.081–1.698)	6.96**	.008
Alternative news media and blogs	0.819 (0.682–0.983)	4.59*	.032
**Topic-specific trust in news sources**			
Federal government	1.133 (0.907–1.415)	1.20	.273
City government	1.026 (0.820–1.285)	0.05	.820
Political parties	0.934 (0.724–1.206)	0.27	.601
Individual politicians	0.910 (0.721–1.150)	0.62	.432
WHO	0.959 (0.756–1.217)	0.12	.729
RKI	0.858 (0.648–1.137)	1.13	.287
National commission on vaccination	1.114 (0.887–1.398)	0.86	.353
National board of ethics	1.162 (0.962–1.403)	2.42	.120
Hospitals	0.875 (0.708–1.081)	1.53	.217
Public health offices	0.907 (0.736–1.117)	0.85	.358
Business and industry associations	1.122 (0.923–1.363)	1.34	.248
Churches	1.215 (1.027–1.438)	5.14*	.023
**Intensity of general media use**			
TV (offline)	1.028 (0.961–1.100)	0.66	.418
Radio (offline)	1.065 (0.986–1.150)	2.55	.110
Print media (offline)	1.002 (0.917–1.095)	0.00	.961
Online media	0.978 (0.804–1.190)	0.05	.827
**Topic-related information seeking**			
Intensity of information seeking	1.023 (0.963–1.088)	0.55	.461
TV (offline) (with use coded high)	1.138 (0.801–1.617)	0.52	.472
Radio (offline) (with use coded high)	1.409 (0.963–2.061)	3.12	.077
Print media (offline) (with use coded high)	1.268 (0.888–1.810)	1.71	.191
Conversations and chats with health professionals (with use coded high)	1.656 (1.138–2.411)	6.95**	.008
Conversations and chats with (other) patients (with use coded high)	1.103 (0.554–2.197)	0.08	.780
Online news sites (with use coded high)	0.866 (0.581–1.291)	0.50	.480
Online TV and video streaming (e.g. Netflix) (with use coded high)	1.257 (0.673–2.347)	0.51	.473
Online audio streaming and podcasts (with use coded high)	1.000 (0.683–1.464)	0.00	.998
Video platforms (e.g., YouTube) (with use coded high)	0.622 (0.449–0.862)	8.15**)	.004
Social media (with use coded high)	1.334 (0.953–1.868)	2.82)	.093
COVID-19 warning app (with use coded high)	1.360 (0.986–1.874)	3.52)	.061

Binary logistic regression analysis. χ^2^(44) = 276.33, *p* < .001, n = 1,114; Nagelkerke R^2^ = .311; Cohen’s f^2^ = . 45.

*p < .05; **p < .01; ***p < .001.

Neither the intensity of topic-related information seeking nor the topic-related use of offline mass media were significantly associated with the likelihood of vaccination. The same was true for the topic-related use of online news sites, video streaming, or audio streaming, social media, conversations and chats with (other) patients, or the use of the COVID-19 warning app. The likelihood of vaccination was 65.6% higher when students reported topic-related conversations and chats with health professionals and it was lower by 37.8% when students reported the topic-related use of video platforms like YouTube.

### Main analysis II: Vaccination intention (unvaccinated students)

Among the unvaccinated students in the final sample (n = 334), 26.9% were male and 70.7% were female, 2.4% specified themselves as diverse or open. The sample was slightly younger (M = 22.9; SD = 3.5). The intention to get vaccinated among unvaccinated students proved to be high: more than two-thirds (67.8%) stated that it was “very likely” that they would get vaccinated if they were offered vaccination against COVID-19, while less than one in ten (7.8%) stated that this was “very unlikely” (M = 9.2; SD = 3.2).

The multiple linear regression model was statistically significant (F(42,291) = 21.32, *p* < 0.001, n = 334) and explained 71.9% of variance in unvaccinated university students’ intention to vaccinate (adjusted R^2^ = 0.719), resulting in a large Cohen’s f effect size of f^2^ = 2.56. Of the variables entered into the regression model after basic analyses, eight were significantly associated with unvaccinated university students’ COVID-19 vaccination intention (Table 3). Specifically, psychological factors and different shades of trust in information sources were associated with unvaccinated university students’ intention to consider vaccination against COVID-19. Regarding the five dimensions of the 5C model, confidence in the safety and effectiveness of vaccination, perception of COVID-19 as a risk, and perception of vaccination as a collective responsibility were positively associated with the intention to vaccinate. No significant association was found in the weighing of risks and benefits and the intention to get vaccinated while there was a slight correlation between emphasizing everyday stress as a barrier for vaccination and the intention to get vaccinated. General media trust and the specific trust in the regional press (including online outlets) were slightly positively associated with the intention to get a vaccination against COVID-19. Topic-specific media trust, and specific trust in public and private broadcasters, national quality press, tabloid media, alternative news media, or online messenger services were not significantly associated with vaccination intention. Topic-related trust in the central German health authority RKI as an information source was slightly positively associated with vaccination intention. Except for the use of the official COVID-19 warning app, general and topic-related media use were not significantly associated with unvaccinated students’ vaccination intention. The same was found for study- and health-related factors, such as field of study and global interest in common vaccinations.

**Table 3:** Associations of sociodemographic, study-related, psychological, health-related, and communication-related factors with the intention to get vaccinated against COVID-19 among unvaccinated university students in the summer of 2021.

	β	T	*p*
**Sociodemographic and study-related factors**			
Field of study (with medicine coded high)	− .04	1.19	.236
**Health-related factors**			
Global interest in common vaccinations	− .02	0.56	.576
**Psychological factors**			
Confidence	.35	7.83***	< .001
Complacency	− .18	3.81***	< .001
Constraints	.07	2.29*	.023
Calculation	− .02	0.72	.470
Collective Responsibility	− .34	7.95***	< .001
**Communication-related factors**			
**Media trust**			
General media trust	.10	2.33*	.020
Topic-specific media trust	− .05	1.07	.287
Public broadcasting (including online outlets)	− .07	1.52	.131
Private broadcasting (including online outlets)	− .06	1.49	.138
National quality press (including online outlets)	− .06	1.37	.173
Regional press (including online outlets)	.10	2.75**	.006
Tabloid media (including online outlets)	− .01	0.14	.890
Alternative news media & blogs	.02	0.47	.642
Online messenger	.04	1.27	.206
**Topic-specific trust in news sources**			
Federal government	− .01	0.10	.922
City government	− .02	0.40	.687
Foreign governments and authorities	− .04	0.99	.324
Political parties	− .00	0.09	.927
Individual politicians	.07	1.69	.091
WHO	− .03	0.59	.554
RKI	.14	2.74**	.007
National commission on vaccination	.01	0.27	.789
National board of ethics	− .06	1.55	.123
Hospitals	− .04	1.01	.315
Doctors	.03	0.80	.426
Pharmacists	.04	0.89	.374
Nurses	.00	0.01	.991
Public health offices	.01	0.26	.795
Health insurance companies	.03	0.66	.507
Universities and scientific institutes	.06	1.27	.205
Local university	− .03	0.70	.482
Unions	.02	0.50	.618
Ordinary people known personally	− .05	1.41	.158
Ordinary people not known personally	− .02	0.49	.628
**Intensity of general media use/information seeking**			
Online media	.03	0.90	.368
Topic-related information seeking	− .03	0.93	.353
**Topic-related information seeking—Sources**			
Online news sites (with use coded high)	− .00	0.09	.932
COVID-19 warning app (with use coded high)	.07	2.02*	.044
Government or agency websites (with use coded high)	.04	1.19	.235
University websites (with use coded high)	.05	1.70	.090

Multiple linear regression analysis. F(42,291) = 21.32, *p* < .001, n = 334; Adjusted R^2^ = .719; Cohen’s f^2^ = 2.56.

*p < .05; **p < .01; ***p < .001.

## Discussion

In order to better understand the determinants of university students’ vaccination behavior during the corona crisis, our study aimed to investigate how *sociodemographic and study-related, health-related, psychological,* and *communication-related* factors were associated with the likelihood of vaccination against COVID-19 among university students and the vaccination intention among students who had not been vaccinated in summer of 2021. Our results show that, similar to other types of vaccinations^4,7,8^, broader populations^19,56,57^, and countries^32^, general psychological determinants for vaccination intentions and behavior also play a major role in COVID-19 vaccination among German university students. Those with higher confidence in vaccination were more likely to get vaccinated against COVID-19, whereas those with lower confidence had a lower likelihood of vaccination. Accordingly, with students’ increasing weighing of benefits and risks, vaccination became less likely. Neither individual risk perception nor one’s own sense of responsibility for community health appeared to be influential factors for vaccination in the summer term of 2021. The latter could be due to the fact that the individual risk for university students to develop severe COVID-19 was comparatively low due to the young age of the target group. Moreover, the overall case numbers in Germany in the summer of 2021 were low.

The subjective perception of vaccination barriers was an important factor for university students’ actual COVID-19 vaccination behavior, a fact that was generally repeatedly observed in Germany in vaccination attitudes and intentions in the past^4,58^. In this special case, however, this could also be due to the special situation at universities and in the German vaccination campaign at that time. Prioritization ended in the middle of the (online) semester, and thus the main effort to obtain a vaccination appointment, as well as the vaccination appointments themselves, fell into a traditionally exam-intensive period. It is important to note that the students were largely on their own in this regard. Coordinated vaccination programs for all students at the university did not generally exist at that time. After vaccination prioritization ended, some students might not have been vaccinated because they could have been stressed by their study workload and exams or have thought that they could not manage waiting in line for hours for a vaccination appointment during the semester, even though they were willing to get vaccinated. It is absolutely in line with this that among the unvaccinated students in the sample, a stronger reporting of subjective barrierers was slightly but significantly associated with the reporting of a higher intention to get vaccinated in the future.

In this context, special attention should also be paid to the fact that being a medical student substantially increased the likelihood of a vaccination. There could be several reasons for this association, some of which could also be related to de facto barriers to vaccination for other students in the summer term of 2021. Medical students, similar to medical staff in general, were prioritized and thus had earlier access to vaccines than other students, according to the restrictive prioritization politics of the German government. They could also have had better and closer contact with and easier access to healthcare professionals who offered vaccinations. Moreover, because of their field of study, they could have been more able to assess the value and importance of vaccination for themselves and others.

In any case, for university students, drawing on health expertise seems to have been beneficial to a positive vaccination decision. Students who had conversations and chats with health professionals on the topic of COVID-19 were more likely to be vaccinated against the disease in the summer of 2021. Conversely, the use of video platforms (e.g., YouTube) as an information source was associated with a lower likelihood of vaccination. The latter could also be related to the fact that the health-related quality of the content on these platforms is heterogeneous and that voices critical of vaccination and conspiracy myths were increasingly observed there^59^. The reception of this type of content was recently found to reduce vaccination intent^27^. This is consistent with the fact that with increasing trust in alternative media and blogs, which mainly provided and spread vaccine-critical content and positions in Germany during the COVID-19 pandemic^60^, the likelihood of vaccination decreased.

By contrast, even if similar content could be found on these platforms^60,61^, social media trust was positively associated with vaccination. This finding implies an important message that is sometimes overlooked in the often-heated social and political debate about the potential (negative) effect of social media on attitudes, intentions, and behavior: social media are not good or bad per se, but channels through which different types of information can be obtained. Therefore, trust in these channels does not necessarily have to have negative effects, but, as in the present case, it may be vaccine promoting if the “right” content is used. As younger audiences’ health-related information use does take place in these channels, health authorities’ efforts should focus on positively influencing the quality of content in these channels.

Topic-related trust in churches was positively related with the likelihood of a vaccination. This seems plausible as the Christian churches in Germany, to which still about half of the German population formally belong, had in fact supported the vaccination campaign and called on members and believers to get vaccinated on various occasions. In this context, it should be noted that the role of social influence has not been included in this study. However, this kind of finding could also be a clue that the social environment and its influences might well be relevant in COVID-19 vaccination. It is known that social norms within a group or society can affect (health) intentions and behavior^62,63^ and were also important in the COVID-19 pandemic^64–68^. Different social norms exist and operate in different social groups, likely also with regard to how a vaccination against COVID-19 is assessed. Depending for example on affiliation to religious communities, family context, or peer groups, this may not only have consequences in expectations and beliefs about the good and bad of vaccination, but result in different intentions to get vaccinated and/or actual vaccination behavior. Furthermore, influences of the social environment are likely to be indirectly effective as well. For example, social environment could shape communication behavior or trust in certain media channels, information sources, and institutions – which can then in turn have an effect on vaccination behavior themselves.

The special focus on unvaccinated students’ intention to get vaccinated against COVID-19 yields important insights into university students’ vaccination decision. The linear regression model showed some similar tendencies but also other factors influencing vaccination intention of those who had not (yet) been vaccinated. Again, confidence in vaccination safety was a strong determinant of vaccination intention among the unvaccinated students, which was positively associated with unvaccinated students’ intention to get vaccinated. As mentioned before, a stronger subjective perception of vaccination barriers was also positively associated with vaccination intention – and not negatively as in earlier findings in other European countries^32^. This could be a sign that there actually were barriers for certain German student populations in the summer of 2021 which prevented them from getting their vaccination (ealier).

Furthermore, both the individual perception of the disease as a risk and the perception of vaccination as a collective responsibility were associated with the intention to get vaccinated. The more the unvaccinated students perceived COVID-19 as a risk and the more they perceived a vaccination against COVID-19 as a collective responsibility, the more likely they stated that they would get vaccinated if they were offered vaccination. Consequently, for future COVID-19 communication campaigns in the target group of rather hesistant students, it could make sense to emphasize both the value of vaccination for themselves and for the community.

The field of study had no influence on unvaccinated students intention to get vaccinated against COVID-19, nor had the general interest in common vaccinations. The latter suggests that the intention to get or not to get vaccinated against COVID-19 among unvaccinated students in summer term 2021 was quite specific to this vaccination and had less to do with a general aversion to or a general support of vaccinations.

In contrast, aspects of trust in (certain) media and institutions proved to be significantly related to vaccination intention. General media trust was positively associated with unvaccinated students intention to get vaccinated, as was trust in regional press media. Topic-related trust in Germany’s central health authority RKI also seemed to have played a decisive role for unvaccinated students and was positively associated with vaccination intention. Accordingly, the use of the official COVID-19 warning app as an information source, which is provided at no cost by the RKI, was slightly positively associated with vaccination intention. These findings are in line with earlier findings in several countries^17,18,22,37,38^, showing that in the very special context of COVID-19 vaccination, general trust in traditional media sources and official health authorities, which in this special case provided serious information clearly in favor of a vaccination, were especially benefitial for a positive vaccination decision, and that this seemed to be especially true for students who initially hesitated with a vaccination against COVID-19. Therefore, also in view of future health crises, it might be useful to explain the work of serious journalistic media and health authorities even better, in order to reduce existing reservations and to strengthen the general trust in these institutions which play a crucial role when it comes to health information during crises of public health.

## Limitations

Our study has some limitations, especially with regard to its student sample and survey design. Only students from a single German university were surveyed. Moreover, the composition of the sample in terms of age and gender differed from the composition of the University’s students body and the basic population of university students in Germany. As the students were free to participate in the survey, self-selection could be a relevant factor. Nevertheless, the proportions of vaccinated and unvaccinated students in the sample were almost the same as those of the relevant age groups reported in surveys representative of the German population at the time of data collection^69^.

Furthermore, our findings were based on cross-sectional data. Even if it is theoretically plausible to assume certain relationships, temporal links between the variables cannot be determined because they were examined at the same time. Therefore, inferences in causality are impossible.

All data, including information on vaccination behavior, were based on the self-reports of the respondents. An observation of the actual individual vaccination process did not take place. For each factor group, only a selection of relevant variables could be included in the survey and analyses. In some cases, some information was asked generally in the survey, so that certain details were not available for analysis. For example, we asked respondents to indicate their information sources from a list of types of services (e.g., social media, online messenger, and video platforms) without specifically asking for concrete services (e.g., Facebook, Instagram, WhatsApp, Telegram, and YouTube). Some latent variables, such as the psychological determinants and media trust, were measured using one-item short scales.

As in most studies focusing on human ideas, attitudes, and behavior, relevant variables are seldom completely uncorrelated; thus, possible multicollinearity should be checked. As to be expected, low to moderate multicollinearity was observed. Still, correlations between the independent variables were low (*r* ≤ 0.65) and VIF showed no values higher than 3.5, indicating that multicollinearity was not a serious confounding factor in the analyses.

These limitations should be taken into account. Nonetheless, our findings provide a good foundation for future studies on the influencing factors of university students’ vaccination.

## Conclusion

Our results indicate that general vaccine-related factors are also important in German university students’ decision to get COVID-19 vaccination, with a high relevance of psychological factors, but also some communication- and study-related factors being important. Greater trust in social media and topic-related conversations and chats with health professionals were associated with a higher likelihood of vaccination, whereas greater trust in alternative news media and the topic-related use of video platforms (e.g., YouTube) were associated with a lower likelihood of vaccination. Furthermore, studying in medical school was associated with a higher likelihood of vaccination. For the students who had not yet been vaccinated against COVID-19 by summer of 2021, in addition to confidence in vaccination safety, the perception of barriers, the perception of the disease as a health risk and the extent to which responsibility for others was perceived or not were also important predictors of vaccination intention.

Confidence in the safety of vaccinations is crucial for vaccination uptake. Thus, for a COVID-19 vaccination campaign for university students to succeed, students should have a discussion with competent health experts and should be supported when assessing health-related media sources and the information they come across. As trust in and the use of certain sources seem to be linked to vaccination behavior and intentions, universities and health authorities should strengthen students’ basic media literacy in the long run, independent of acute pandemic situations or ongoing vaccination campaigns. To convince those who have not been vaccinated, but are basically reachable, emphasizing the risks of COVID-19 and the responsibility that one has for the sake of others by getting vaccinated is an important argument.

## Supplementary Information

Supplementary Information.

## Data Availability

The dataset generated and analyzed in the current study is stored in the server of the University Medical Center of the JGU Mainz (European server) and is available from the corresponding author upon reasonable request.
