# Supplementary material for: Determinants of university students’ COVID-19 vaccination intentions and behavior
Source: Sci Rep. 2022 Oct 27;12:18067. doi: 10.1038/s41598-022-23044-9 (PMC9610342; doi:10.1038/s41598-022-23044-9)
Supplement: Supplementary file 1 — Supplementary Information. [file 41598_2022_23044_MOESM1_ESM.pdf]

Determinants of university students’ COVID-19 vaccination intentions and behavior

Supplemental Material

Supplementary Table 1

Items used

**Dimension**<br>Variable	**Nr. Items**	**Items**,<br>*answering options*
Dependent variables		
Vaccination status	1	“Have you already been vaccinated against COVID-19?”<br>*0 = ”no”, 1 = “yes, once”, 2 = “yes, twice”;*<br><br>*answers recoded for analysis:*<br>*0 = no vaccination, 1 = at least one vaccination*
Vaccination willingness	1	“How likely is it that you will get vaccinated if you are offered vaccination against COVID-19?”<br>*1 = „very unlikely“, 11 = „very likely“*
Independent variables		
Sociodemographic and study-related		
Age	1	*Open, in years*
Gender	1	*male, female, diverse, open*
Field of study	1	“Please select your (main) field of study from the list below. If you are studying more than one subject, please indicate the field you consider to be the main focus of your studies.”<br><br>*List of all available fields of study at the corresponding university; answers recoded for analysis:*<br>*1 = medicine; 0 = other fields of study*
Health-related		
General health status	1	“If you rate the best conceivable state of health with 10 points and the worst conceivable with 0 points, how many points would you award your current state of health?”<br>*0 = “worst conceivable state of health”*<br>*10 = “best conceivable state of health”*
Well-being (WHO-5)	5	“I have felt cheerful and in good spirits”<br>“I have felt calm and relaxed”<br>“I have felt active and vigorous”<br>“I woke up feeling fresh and rested”<br>“My daily life has been filled with things that interest me”)<br><br>*0 = ”None of the time”*<br>*1 = “Some of the time”*<br>*2 = “Less than half the time”*<br>*3 = “More than half the time”*<br>*4 = “Most of the time”*<br>*5 = “All of the time”*
Chronic disease	1	“Have you been diagnosed with a chronic disease?”

		0 = „no“, 1 = „yes“
Disability	1	“Have you been diagnosed with a disability?“<br>*0 = „no“, 1 = „yes“*
Health literacy	4	“How easy/difficult is it to...<br>a) …find information about symptoms of illness that affect you?”<br>b) …understand what to do in a medical emergency?”<br>c) …judge when you should see a health professional for a check-up?”<br>d) …make decisions that will improve your health?”<br><br>*0 = “very difficult”*<br>*1 = “fairly difficult”*<br>*2 = “fairly easy”*<br>*3 = „very easy“*
General interest in common vaccinations	1	“How important is it to you to have adequate vaccination protection against common diseases (e.g., mumps, measles, rubella, and tetanus)?”<br>*1 = “not important at all”, 5 = “very important”*
Psychological		
Confidence	1	“I am completely confident that vaccination against COVID-19 is safe.”<br>*1 = “do not agree at all”, 7 = “fully agree”*
Complacency	1	“Vaccination is unnecessary because COVID-19 is not a major threat.”<br>*1 = “do not agree at all”, 7 = “fully agree”*
Constraints	1	“Everyday stress prevents me from getting vaccinated against COVID-19.”<br>*1 = “do not agree at all”, 7 = “fully agree”*
Calculation	1	“When I think about getting vaccinated against COVID-19, I weigh the benefits and risks to make the best decision possible.”<br>*1 = “do not agree at all”, 7 = “fully agree”*
Collective responsibility	1	“When everyone is vaccinated against COVID-19, I don’t have to get vaccinated, too.”<br>*1 = “do not agree at all”, 7 = “fully agree”*
Communication-related		
General media trust	1	“In general terms, how much do you think the media in Germany can be trusted?”<br>*1 = “not at all”, 2 = “rather not”, 3 = “partly”, 4 = “rather”, 5 = “completely”*
Topic-specific media trust	1	“There is currently a lot of media coverage of the COVID-19 pandemic. How much can the media in Germany be trusted on this topic?”<br>*1 = “not at all”, 2 = “rather not”, 3 = “partly”, 4 = “rather”, 5 = “completely”*
Trust in specific media sources	1	“Some people consider certain media sources more trustworthy than others. How about you? Please indicate how trustworthy you think the following media sources are.”<br><br>• *Public broadcasting (including online outlets)*<br>• *Private broadcasting (including online outlets)*<br>• *National press media (including online outlets)*

		• *Regional press media (including online outlets)*<br>• *Tabloid media (including online outlets)*<br>• *Alternative news media and blogs*<br>• *Online portals*<br>• *Social media*<br>• *Online messenger*<br>• *Video platforms*<br><br>*1 = "not at all", 2 = "rather not", 3 = "partly", 4 = "rather", 5 = "very"*
Topic-related trust in news sources	1	"How trustworthy are the following sources with regard to the coronavirus topic?"<br><br>• *German federal government*<br>• *State government*<br>• *City*<br>• *Foreign governments and authorities*<br><br>• *World Health Organization (WHO)*<br>• *Robert Koch Institute (RKI)*<br>• *National commission on vaccination*<br>*National Board of Ethics*<br><br>• *Public health offices*<br>• *Hospitals*<br>• *Doctors*<br>• *Pharmacists*<br>• *Nurses*<br>• *Health insurance companies*<br><br>• *Political parties*<br>• *Individual politicians*<br><br>• *Universities and scientific institutes*<br>• *Local university*<br>• *Individual scientists*<br>• *Unions*<br>• *Business and industry associations*<br>• *Churches*<br><br>• *Ordinary people I know personally*<br>• *Ordinary people I don't know personally*<br><br>*1 = "not at all", 2 = "rather not", 3 = "partly", 4 = "rather", 5 = "very"*
Intensity general media use	4	*Recorded in days per week (0–7):*<br><br>1.) "How often do you use the following media?"<br><br>*Response options: "Never" (= 0), "Less often than once a month" (= 0), "At least once a month" (= 0), "At least once a week," "At least once a day" (= 7).*

		If used "at least once a week":<br><br>2.) "Now, in terms of an ordinary week, how many days a week do you watch TV/listen to the radio/read newspapers and magazines/use the Internet?"<br><br>*0–7 days*
Intensity topic-related information seeking	1	"Some people gather information about the coronavirus topic on a daily basis, whereas others do not. We are interested in how it is with you. How often do you gather information on this topic?"<br><br>*Response options: "Never" (= 0), "Less often than once a month" (= 0), "At least once a month" (= 0). "At least once a week," "At least once a day" (= 7).*<br><br>*If used "at least once a week":*<br><br>2.) "Now, in terms of an ordinary week, how many days a week do you gather information on the coronavirus topic?"<br><br>*0–7 days*
Topic-related information sources	2	"Where have you gathered information on the coronavirus topic during the past 12 months?"<br><br>• *Internet*<br><br>• *TV (offline)*<br>• *Radio (offline)*<br>• *Journalistic print media (offline)*<br><br>• *Books, health guides, encyclopedias*<br>• *Free brochures or magazines from health insurance companies, pharmacies, or other providers (offline)*<br><br>• *Face-to-face conversations or chats with family members, friends, and colleagues*<br>• *Face-to-face conversations or chats with doctors, therapists, and nurses*<br>• *Face-to-face conversations or chats with pharmacists*<br>• *Face-to-face conversations or chats with other patients or affected persons*<br><br>• *Counseling centers, health or educational institutions*<br>• *Telephone (counseling) services offered by health insurance companies, patient or consumer protection organizations*<br><br>• *Other sources*

		Multiple answers possible; 0 = no use, 1 = use<br><br>“Which online sources have you used for gathering information on the coronavirus topic?”<br><br>• *Online news sites*<br>• *Online TV and video streaming*<br>• *Online radio, audio streaming, and podcasts*<br>• *Video platforms (e.g., YouTube)*<br><br>• *Search engines*<br>• *Social media*<br>• *Online messenger*<br><br>• *Service communities*<br>• *Health portals (e.g., netdoktor, onmedia)*<br>• *Blogs on the topic of health and disease*<br>• *Health forums and communities*<br>• *Comparison portals for searching doctors, hospitals, and nursing homes*<br><br>• *Government or agency websites*<br>• *COVID-19 warning app*<br>• *University websites*<br><br>• *Medical online consultation*<br>• *Websites of doctors, hospitals, rehabilitation or care facilities*<br>• *Websites of health or patient organizations*<br>• *Online pharmacies*<br>• *Websites of health insurance companies*<br><br>• *Other sources*<br><br>*Multiple answers possible; 0 = no use, 1 = use*

Basic analyses

Vaccination behavior

Sociodemographic and study-related factors

Supplementary Table 2

Gender differences between vaccinated and unvaccinated university students

	Vaccinated<br>n=974<br>%	Unvaccinated<br>n=424<br>%
male	22.7	25.7
female	74.9	71.9
diverse	0.8	0.9
open	1.5	1.4

$\chi^2(3) = 1.58, p = .663$.

Supplementary Table 3

Age differences between vaccinated and unvaccinated university students

	Vaccinated<br>n=971<br>M (SD)	Unvaccinated<br>N=423<br>M (SD)	t (df)	p
Age	23.92 (4.97)	23.16 (4.10)	2.75 (1392)	.006

Supplementary Table 4

Differences in field of study between vaccinated and unvaccinated university students

	Vaccinated<br>n=918<br>%	Unvaccinated<br>n=409<br>%
Medicine	19.3	7.1
Other fields	80.7	92.9 %

$\chi^2(1) = 32.06, p < .001$.

Health-related factors

Supplementary Table 5

Differences in general health status between vaccinated and unvaccinated university students

	Vaccinated<br>n=931<br>M (SD)	Unvaccinated<br>n=398<br>M (SD)	t (df)	p
General health status	6.91 (1.89)	6.69 (2.05)	1.83 (697.795)	.067

Supplementary Table 6

Differences in well-being between vaccinated and unvaccinated university students

	Vaccinated n=908 M (SD)	Unvaccinated n=388 M (SD)	t (df)	p
Well-being (WHO-5)	11.56 (5.40)	11.01 (5.50)	1.63 (1294)	.100

Supplementary Table 7

Differences in the presence/absence of chronic diseases and/or disability between unvaccinated university students

Chronic diseases/ Disability	Vaccinated n=930 %	Unvaccinated n=397 %
yes	2.2	1.0
no	97.8	99.0

$\chi^2(1) = 2.05, p = .153$;

Supplementary Table 8

Differences in health literacy between vaccinated and unvaccinated university students

	Vaccinated n=957 M (SD)	Unvaccinated n=418 M (SD)	t (df)	p
Health literacy	7.89 (2.13)	7.47 (2.19)	3.35 (1373)	.001

Suppl. Table 9

Differences in general interest in common vaccinations (e.g., mumps, measles, rubella, tetanus) between vaccinated and unvaccinated university students

	Vaccinated n=970 M (SD)	Unvaccinated n=416 M (SD)	t (df)	p
General interest in common vaccinations	4.61 (0.68)	4.39 (0.89)	4.56 (629.479)	<.001

Psychological factors

Supplementary Table 10

Differences in confidence between vaccinated and unvaccinated university students

	Vaccinated n=970 M (SD)	Unvaccinated n=416 M (SD)	t (df)	p
Confidence	5.68 (1.14)	4.74 (1.76)	10.00 (569.650)	<.001

Supplementary Table 11

Differences in complacency between vaccinated and unvaccinated university students

	Vaccinated n=967 M (SD)	Unvaccinated n=415 M (SD)	t (df)	p
Complacency	1.28 (0.81)	1.77 (1.35)	6.88 (546.523)	<.001

Supplementary Table 12

Differences in constraints between vaccinated and unvaccinated university students

	Vaccinated n=969 M (SD)	Unvaccinated n=416 M (SD)	t (df)	p
Constraints	1.21 (0.70)	1.84 (1.38)	8.85 (507.843)	<.001

Supplementary Table 13

Differences in calculation between vaccinated and unvaccinated university students

	Vaccinated n=969 M (SD)	Unvaccinated n=416 M (SD)	t (df)	p
Calculation	4.39 (1.94)	4.88 (1.83)	4.46 (828.275)	<.001

Supplementary Table 14

Differences in collective responsibility between vaccinated and unvaccinated university students

	Vaccinated n=969 M (SD)	Unvaccinated n=415 M (SD)	t (df)	p
Collective Responsibility	1.38 (0.97)	2.12 (1.65)	8.54 (540.081)	<.001

Communication-related factors

Supplementary Table 15

Differences in general media trust between vaccinated and unvaccinated university students

	Vaccinated n=956 M (SD)	Unvaccinated n=412 M (SD)	t (df)	p
General media trust	2.89 (0.84)	2.77 (0.92)	2.31 (719.322)	.021

Supplementary Table 16

Differences in topic- specific media trust between vaccinated and unvaccinated university students

	Vaccinated n=959 M (SD)	Unvaccinated n=409 M (SD)	t (df)	p
Topic-specific media trust	3.32 (0.81)	3.16 (0.90)	3.12 (1366)	.002

Supplementary Table 17

Differences in trust in specific media sources between vaccinated and unvaccinated university students

	Vaccinated M (SD)	Unvaccinated M (SD)	t (df)	p
Public broadcasting (including online outlets)	4.39 (0.84) *n=954*	4.12 (1.02) *n=408*	4.68 (655.467)	<.001
Private broadcasting (including online outlets)	3.02 (0.91) *n=951*	2.86 (0.95) *n=407*	2.76 (740.366)	.006
National press media (including online outlets)	4.13 (0.86) *n=954*	3.95 (0.90) *n=408*	3.44 (1360)	.001
Regional press media (including online outlets)	3.70 (0.84) *n=950*	3.60 (0.82) *n=406*	1.93 (1354)	.054
Tabloid media (including online outlets)	1.70 (0.79) *n=953*	1.69 (0.79) *n=408*	0.32 (1359)	.753
Online portals (e.g., *t-online.de, gmx.de*)	2.47 (0.88) *n=950*	2.50 (0.88) *n=404*	0.57 (1352)	.569
Alternative news media & blogs	2.10 (0.92) *n=944*	2.31 (0.90) *n=406*	3.96 (1348)	<.001
Video platforms (e.g. *YouTube*)	2.55 (0.82) *n=953*	2.61 (0.81) *n=408*	1.16 (1359)	.245
Social media (e.g., *Facebook, Instagram*)	2.15 (0.82) *n=953*	1.98 (0.77) *n=409*	3.71 (818.855)	<.001
Messenger services (e.g., *Whatsapp, Telegram*)	1.68 (0.78) *n=954*	1.71 (0.78) *n=408*	0.78 (1360)	.436

Suppl. Table 18

Differences in topic-specific trust in news sources between vaccinated and unvaccinated university students

	Vaccinated M (SD)	Unvaccinated M (SD)	t (df)	p
German federal government	3.57 (0.94) *n=941*	3.25 (1.04) *n=401*	5.42 (1340)	<.001
State government	3.61 (0.90) *n=940*	3.37 (0.98) *n=402*	4.37 (1340)	<.001
City	3.67 (0.84) *n=943*	3.49 (0.88) *n=402*	3.60 (1343)	<.001
Foreign governments and	2.88 (0.72)	2.88 (0.78)	0.01	.991

authorities	*n=940*	*n=401*	(1339)	
World Health Organization (WHO)	4.37 (0.85) *n=939*	4.10 (1.06) *n=402*	4.50 (631.340)	<.001
Robert Koch Institute (RKI)	4.58 (0.79) *n=942*	4.32 (1.01) *n=402*	4.60 (616.514)	<.001
National commission on vaccination (STIKO)	4.31 (0.86) *n=940*	3.93 (1.06) *n=403*	6.39 (639.647)	<.001
National board of ethics	3.66 (0.98) *n=941*	3.35 (0.98) *n=401*	5.29 (1340)	<.001
Public health offices	3.99 (0.91) *n=944*	3.82 (1.05) *n=403*	2.85 (669.459)	.005
Doctors	4.31 (0.80) *n=944*	4.26 (0.81) *n=403*	1.01 (1345)	.311
Hospitals	4.15 (0.85) *n=939*	4.02 (0.92) *n=401*	2.46 (1338)	.014
Pharmacists	4.03 (0.80) *n=944*	3.94 (0.90) *n=403*	1.78 (1345)	.075
Nurses	3.89 (0.90) *n=943*	3.93 (0.92) *n=402*	0.72 (1343)	.472
Health insurance companies	3.61 (0.88) *n=941*	3.53 (1.03) *n=402*	1.44 (667.188)	.151
Universities and scientific institutes	4.40 (0.79) *n=941*	4.32 (0.82) *n=401*	1.79 (1340)	.074
Individual scientists	3.61 (0.85) *n=940*	3.57 (0.87) *n=402*	0.83 (1340)	.408
Local University	4.11 (0.85) *n=940*	4.01 (0.92) *n=401*	1.89 (1339)	.059
Political parties	2.69 (0.78) *n=941*	2.52 (0.79) *n=403*	3.55 (1342)	<.001
Individual politicians	2.69 (0.78) *n=944*	2.58 (0.84) *n=400*	2.41 (702.372)	.016
Unions	3.02 (0.78) *n=941*	2.98 (0.77) *n=400*	0.84 (1339)	.401
Business and industry associations	2.65 (0.88) *n=944*	2.52 (0.87 *n=402*	2.36 (1344)	.018
Churches	2.26 (1.03) *n=940*	2.10 (1.03) *n=402*	2.56 (1340)	.011
Ordinary people known personally	3.05 (0.85) *n=944*	3.00 (0.88) *n=403*	1.00 (1345)	.317
Ordinary people not known personally	2.05 (0.87) *n=941*	2.04 (0.84) *n=403*	0.24 (1342)	.810

Supplementary Table 19

Differences in intensity of general media use between vaccinated and unvaccinated university students

	Vaccinated M (SD)	Unvaccinated M (SD)	t (df)	p
TV (offline)	1.88 (2.65) *n=948*	1.49 (2.57) *n=403*	2.53 (779.243)	.012
Radio (offline)	1.80 (2.55) *n=935*	1.21 (2.20) *n=402*	4.28 (872.622)	<.001
Newspapers & magazines (offline)	1.17 (2.21) *n=947*	0.82 (1.89) *n=403*	3.00 (880.062)	.003
Internet	6.89 (0.80) *n=949*	6.77 (1.14) *n=404*	1.99 (579.654)	.047

Supplementary Table 20

Differences in intensity of topic-related information seeking between vaccinated and unvaccinated university students

	Vaccinated n=974 M (SD)	Unvaccinated *n=424* M (SD)	t (df)	p
Topic-related information seeking	4.03 (2.58)	3.25 (2.70)	5.12 (1396)	<.001

Supplementary Table 21

Differences in use of topic-related information sources between vaccinated and unvaccinated university students

	Vaccinated (n=974) %	Unvaccinated (n=424) %	$\chi^2(1)$	p
Internet	95.6	92.7	4.94	.026
TV (offline)	64.2	52.1	17.94	<.001
Radio (offline)	46.4	33.0	21.69	<.001
Newspapers & magazines (offline)	43.1	32.8	13.16	<.001
Books, health guides, encyclopedias (offline)	7.9	5.2	3.31	.069
Free brochures or magazines from health insurance companies, pharmacies or other providers (offline)	9.2	10.8	0.87	.351
Face-to-face conversations or chats with family members, friends, colleagues	79.4	75.0	3.28	.070

Face-to-face conversations or chats with doctors, therapists and nurses	31.5	18.4	25.50	<.001
Face-to-face conversations or chats with pharmacists	4.5	2.6	2.89	.089
Face-to-face conversations or chats with other patients or affected persons	7.5	4.0	5.96	.015
Counseling centers, health or educational institutions	8.9	7.5	0.73	.394
Telephone (counseling) services offered by health insurance companies, patient or consumer protection organizations	2.7	1.9	0.76	.383
Other sources	8.7	10.8	1.57	.211

Supplementary Table 22

Differences in use of topic-related online information sources between vaccinated and unvaccinated university students

	Vaccinated (n=931) %	Unvaccinated (n=393) %	$\chi^2(1)$	p
Online news sites	82.3	77.4	4.32	.038
Online radio, audio streaming & podcast	26.9	21.1	4.83	.028
Online TV & video streaming	9.3	5.9	4.43	.035
Video platforms (e.g., *YouTube*)	34.0	39.7	3.84	.050
Search engines	65.0	62.6	0.69	.407
Social media (e.g. *Facebook, Instagram*)	51.3	39.2	16.37	<.001
Online messenger (e.g., *WhatsApp, Telegram*)	19.3	19.8	0.05	.829
Government or agency websites	77.0	73.5	1.83	.176
COVID-19 warning app	59.5	38.4	49.34	<.001
University websites	47.9	48.9	0.10	.752
Online encyclopedia (e.g., *Wikipedia*)	15.9	15.3	0.08	.774
Service communities (e.g., *gutefrage*)	1.3	0.9	0.49	.486
Health portals (e.g., *netdoktor*, *onmedia*)	14.6	13.7	0.17	.681
Health forums and communities	4.4	5.3	0.55	.460
Comparison portals for searching doctors, hospitals and nursing homes	2.3	2.3	0.00	.969
Blogs on the topic of health and disease	6.2	7.6	0.88	.349
Medical online consultation	1.4	1.0	0.31	.576
Websites of doctors, hospitals, rehabilitation or care facilities	13.7	16.3	1.43	.231

Websites of health or patient organizations	11.4	11.7	0.03	.868
Online pharmacies	3.2	3.3	0.01	.936
Websites of health insurance companies	8.6	9.4	0.23	.630
Other online sources	3.0	3.3	0.08	.773

Vaccination intention (unvaccinated students)

Sociodemographic and study-related factors

Supplementary Table 23

Differences in vaccination intention regarding gender

	Male<br>n=109<br>M (SD)	Female<br>n=305<br>M (SD)	Open/diverse<br>n=10<br>M (SD)	F (df)	p
Vaccination intention	9.19 (3.06)	8.82 (3.54)	10.00 (2.49)	0.98 (2, 421)	.378

Supplementary Table 24

Bivariate correlations between vaccination intention and age (n=423)

		r	p
Age	Vaccination intention	-.08	.094

Supplementary Table 25

Differences in vaccination intention regarding field of study

	Medicine<br>n=29<br>M (SD)	Other fields<br>n=380<br>M (SD)	t (df)	p
Vaccination intention	7.52 (3.67)	9.01 (3.39)	2.28 (407)	.023

Health-related factors

Supplementary Table 26

Bivariate correlations between vaccination intention and health-related variables

		r	p
General health status (n=398)	Vaccination intention	-.05	.293
General well-being (n=388)	Vaccination intention	-.06	.242
Health literacy (n=418)	Vaccination intention	.02	.664
General interest in common vaccinations (e.g., mumps, measles, rubella, tetanus) (n=416)	Vaccination intention	.27	<.001

Supplementary Table 27

Differences in vaccination intention regarding the presence/absence of a disability/chronic disease

	Disability/ Chronic Disease n=4 M (SD)	No Disability/ Chronic Disease n=393 M (SD)	t (df)	p
Vaccination intention	7.50 (4.73)	9.12 (3.31)	0.97 (395)	.332

Psychological factors

Supplementary Table 28

Bivariate correlations between vaccination intention and psychological variables

		r	p
Confidence (n=416)	Vaccination intention	.75	<.001
Complacency (n=415)	Vaccination intention	-.70	<.001
Constraints (n=416)	Vaccination intention	-.13	.007
Calculation (n=416)	Vaccination intention	-.31	<.001
Collective responsibility (n=415)	Vaccination intention	-.70	<.001

Communication-related factors

Supplementary Table 29

Bivariate correlations between vaccination intention and communication-related variables – Trust in media sources

		r	p
General media trust (n=412)	Vaccination intention	.39	<.001
Topic- specific media trust (n=409)	Vaccination intention	.47	<.001
Trust in specific media sources			
Public broadcasting (including online outlets) (n=408)	Vaccination intention	.50	<.001
Private broadcasting (including online outlets) (n=407)	Vaccination intention	.21	<.001
National press media (including online outlets) (n=408)	Vaccination intention	.32	<.001
Regional press media (including online outlets) (n=406)	Vaccination intention	.28	<.001
Tabloid media (including online outlets) (n=408)	Vaccination intention	-.11	.027
Online portals (*e.g., t-online.de, gmx.de*) (n=404)	Vaccination intention	.05	.320
Alternative news media & blogs (n=406)	Vaccination intention	-.26	<.001
Video platforms (e.g. YouTube) (n=409)	Vaccination intention	-.08	.129
Social media (e.g., Facebook, Instagram) (n=409)	Vaccination intention	-.02	.689
Messenger services (e.g., Whatsapp, Telegram) (n=408)	Vaccination intention	-.16	.001

Supplementary Table 30

Bivariate correlations between vaccination intention and communication-related variables – Topic-specific trust in news sources

		r	p
German federal government (n=401)	Vaccination intention	.43	.012
State government (n=402)	Vaccination intention	.42	<.001
City (n=402)	Vaccination intention	.31	<.001
Foreign governments and authorities (n=401)	Vaccination intention	.14	.006
World Health Organization (WHO) (n=402)	Vaccination intention	.44	<.001
Robert Koch Institute (RKI) (n=402)	Vaccination intention	.61	<.001
National commission on vaccination (STIKO) (n=403)	Vaccination intention	.51	<.001
National board of ethics (n=401)	Vaccination intention	.22	<.001
Public health offices (n=403)	Vaccination intention	.47	<.001
Doctors (n=403)	Vaccination intention	.39	<.001
Hospitals (n=401)	Vaccination intention	.38	<.001
Pharmacists (n=403)	Vaccination intention	.36	<.001
Nurses (n=402)	Vaccination intention	.18	<.001
Health insurance companies (n=402)	Vaccination intention	.34	<.001
Universities and scientific institutes (n=401)	Vaccination intention	.37	<.001
Individual scientists (n=402)	Vaccination intention	.09	.088
Local University (n=401)	Vaccination intention	.34	<.001
Political parties (n=403)	Vaccination intention	.32	<.001
Individual politicians (n=400)	Vaccination intention	.17	.001
Unions (n=400)	Vaccination intention	.21	<.001
Business and industry associations (n=402)	Vaccination intention	.02	.718
Churches (n=402)	Vaccination intention	-.05	.364
Ordinary people known personally (n=403)	Vaccination intention	-.11	.032
Ordinary people not known personally (n=403)	Vaccination intention	-.12	<.001

Supplementary Table 31

Bivariate correlations between vaccination intention and communication-related variables – Intensity of media use and information-seeking

		r	p
Intensity of general TV use (offline) (n=403)	Vaccination intention	-.05	.313
Intensity of general radio use (offline) (n=402)	Vaccination intention	-.02	.668
Intensity of general use of newspapers & magazines (offline) (n=403)	Vaccination intention	-.02	.728
Intensity of general internet use (n=404)	Vaccination intention	.24	<.001
Intensity of topic-related information seeking (n=424)	Vaccination intention	.12	.014

Supplementary Table 32

Differences in vaccination intention regarding the use/non-use of topic-related information sources - Internet

	Use<br>n=393<br>M (SD)	No use<br>n=31<br>M (SD)	t (df)	p
Vaccination intention	9.05 (3.32)	9.12 (4.18)	1.87 (33.055)	.071

Supplementary Table 33

Differences in vaccination intention regarding the use/non-use of topic-related information sources – TV (offline)

	Use<br>n=221<br>M (SD)	No use<br>n=203<br>M (SD)	t (df)	p
Vaccination intention	9.03 (3.31)	8.85 (3.51)	0.53 (422)	.598

Supplementary Table 34

Differences in vaccination intention regarding the use/non-use of topic-related information sources – Radio (offline)

	Use<br>n=140<br>M (SD)	No use<br>n=284<br>M (SD)	t (df)	p
Vaccination intention	8.96 (3.42)	8.94 (3.40)	0.06 (422)	.954

Supplementary Table 35

Differences in vaccination intention regarding the use/non-use of topic-related information sources – Newspapers & magazines (offline)

	Use<br>n=139<br>M (SD)	No use<br>n=285<br>M (SD)	t (df)	p
Vaccination intention	9.30 (3.21)	8.77 (3.48)	1.56 (294.499)	.120

Supplementary Table 36

Differences in vaccination intention regarding the use/non-use of topic-related information sources – Books, health guides, encyclopedias (offline)

	Use<br>n=22<br>M (SD)	No use<br>n=402<br>M (SD)	t (df)	p
Vaccination intention	8.36 (3.82)	8.98 (3.38)	0.82 (422)	.412

Supplementary Table 37

Differences in vaccination intention regarding the use/non-use of topic-related information sources – Free brochures or magazines from health insurance companies, pharmacies or other providers (offline)

	Use<br>n=46<br>M (SD)	No use<br>n=378<br>M (SD)	t (df)	p
Vaccination intention	9.65 (2.51)	8.86 (3.49)	1.94 (68.301)	.057

Supplementary Table 38

Differences in vaccination intention regarding the use/non-use of topic-related information sources – Telephone (counseling) services offered by health insurance companies, patient or consumer protection organizations

	Use<br>n=8<br>M (SD)	No use<br>n=416<br>M (SD)	t (df)	p
Vaccination intention	9.88 (2.23)	8.93 (3.42)	0.78 (422)	.435

Supplementary Table 39

Differences in vaccination intention regarding the use/non-use of topic-related information sources – Counseling centers, health or educational institutions

	Use<br>n=32<br>M (SD)	No use<br>n=392<br>M (SD)	t (df)	p
Vaccination intention	9.06 (3.08)	8.93 (3.43)	0.21 (422)	.837

Supplementary Table 40

Differences in vaccination intention regarding the use/non-use of topic-related information sources – Face-to-face conversations or chats with other patients or affected persons

	Use<br>n=17<br>M (SD)	No use<br>n=407<br>M (SD)	t (df)	p
Vaccination intention	8.76 (3.29)	8.95 (3.41)	0.22 (422)	.825

Supplementary Table 41

Differences in vaccination intention regarding the use/non-use of topic-related information sources – Face-to-face conversations or chats with family members, friends, colleagues

	Use<br>n=318<br>M (SD)	No use<br>n=106<br>M (SD)	t (df)	p
Vaccination intention	8.97 (3.43)	8.86 (3.35)	0.30 (422)	.767

Supplementary Table 42

Differences in vaccination intention regarding the use/non-use of topic-related information sources – Face-to-face conversations or chats with doctors, therapists and nurses

	Use<br>n=78<br>M (SD)	No use<br>n=346<br>M (SD)	t (df)	p
Vaccination intention	8.44 (3.75)	9.06 (3.32)	1.35 (105.773)	.180

Supplementary Table 43

Differences in vaccination intention regarding the use/non-use of topic-related information sources – Face-to-face conversations or chats with pharmacists

	Use<br>n=11<br>M (SD)	No use<br>n=413<br>M (SD)	t (df)	p
Vaccination intention	9.82 (2.99)	8.92 (3.41)	0.86 (422)	.388

Supplementary Table 44

Differences in vaccination intention regarding the use/non-use of topic-related information sources – Other (offline) sources

	Use<br>n=46<br>M (SD)	No use<br>n=378<br>M (SD)	t (df)	p
Vaccination intention	8.83 (3.31)	8.96 (3.42)	0.25 (422)	.805

Supplementary Table 45

Differences in vaccination intention regarding the use/non-use of topic-related online information sources – Online pharmacies

	Use<br>n=13<br>M (SD)	No use<br>n=380<br>M (SD)	t (df)	p
Vaccination intention	8.38 (3.31)	9.07 (3.42)	0.73 (391)	.464

Supplementary Table 46

Differences in vaccination intention regarding the use/non-use of topic-related online information sources – Service communities (e.g., *gutefrage*)

	Use<br>n=5<br>M (SD)	No use<br>n=388<br>M (SD)	t (df)	p
Vaccination intention	8.20 (4.09)	9.06 (3.31)	0.58 (391)	.566

Supplementary Table 47

Differences in vaccination intention regarding the use/non-use of topic-related online information sources – Websites of doctors, hospitals, rehabilitation or care facilities

	Use<br>n=64<br>M (SD)	No use<br>n=329<br>M (SD)	t (df)	p
Vaccination intention	8.98 (3.29)	9.06 (3.33)	0.17 (391)	.866

Supplementary Table 48

Differences in vaccination intention regarding the use/non-use of topic-related online information sources – Health portals (e.g., *netdoktor, onmedia*)

	Use<br>n=54<br>M (SD)	No use<br>n=339<br>M (SD)	t (df)	p
Vaccination intention	9.44 (2.64)	8.99 (3.41)	1.14 (84.046)	.259

Supplementary Table 49

Differences in vaccination intention regarding the use/non-use of topic-related online information sources – University websites

	Use<br>n=192<br>M (SD)	No use<br>n=201<br>M (SD)	t (df)	p
Vaccination intention	9.40 (2.97)	8.72 (3.60)	2.05 (383.142)	.041

Supplementary Table 50

Differences in vaccination intention regarding the use/non-use of topic-related online information sources – Government or agency websites

	Use<br>n=289<br>M (SD)	No use<br>n=104<br>M (SD)	t (df)	p
Vaccination intention	9.45 (2.96)	7.94 (3.96)	3.54 (146.616)	.001

Supplementary Table 51

Differences in vaccination intention regarding the use/non-use of topic-related online information sources – COVID-19 warning app

	Use<br>n=151<br>M (SD)	No use<br>n=242<br>M (SD)	t (df)	p
Vaccination intention	10.64 (1.15)	8.05 (3.81)	9.88 (306.191)	<.001

Supplementary Table 52

Differences in vaccination intention regarding the use/non-use of topic-related online information sources – Websites of health insurance companies

	Use<br>n=37<br>M (SD)	No use<br>n=356<br>M (SD)	t (df)	p
Vaccination intention	9.57 (2.84)	8.99 (3.36)	1.15 (47.124)	.258

Supplementary Table 53

Differences in vaccination intention regarding the use/non-use of topic-related online information sources – Online encyclopedia (e.g., *Wikipedia*)

	Use<br>n=60<br>M (SD)	No use<br>n=333<br>M (SD)	t (df)	p
Vaccination intention	9.60 (2.65)	8.95 (3.42)	1.67 (98.114)	.098

Supplementary Table 54

Differences in vaccination intention regarding the use/non-use of topic-related online information sources – Health forums and communities

	Use<br>n=21<br>M (SD)	No use<br>n=372<br>M (SD)	t (df)	p
Vaccination intention	8.00 (3.82)	9.11 (3.28)	1.49 (391)	.137

Supplementary Table 55

Differences in vaccination intention regarding the use/non-use of topic-related online information sources – Comparison portals for searching doctors, hospitals and nursing homes

	Use<br>n=9<br>M (SD)	No use<br>n=384<br>M (SD)	t (df)	p
Vaccination intention	9.78 (1.79)	9.03 (3.35)	0.67 (391)	.505

Supplementary Table 56

Differences in vaccination intention regarding the use/non-use of topic-related online information sources – Social media (e.g. *Facebook, Instagram*)

	Use<br>n=154<br>M (SD)	No use<br>n=239<br>M (SD)	t (df)	p
Vaccination intention	8.79 (3.63)	9.21 (3.10)	1.19 (289.377)	.236

Supplementary Table 57

Differences in vaccination intention regarding the use/non-use of topic-related online information sources – Blogs on the topic of health and disease

	Use<br>n=30<br>M (SD)	No use<br>n=363<br>M (SD)	t (df)	p
Vaccination intention	8.03 (3.98)	9.13 (3.25)	1.47 (32.279)	.150

Supplementary Table 58

Differences in vaccination intention regarding the use/non-use of topic-related online information sources – Websites of health or patient organizations

	Use<br>n=46<br>M (SD)	No use<br>n=347<br>M (SD)	t (df)	p
Vaccination intention	9.17 (3.25)	9.03 (3.33)	0.27 (391)	.785

Supplementary Table 59

Differences in vaccination intention regarding the use/non-use of topic-related online information sources – Search engines

	Use<br>n=246<br>M (SD)	No use<br>n=147<br>M (SD)	t (df)	p
Vaccination intention	9.14 (3.19)	8.90 (3.53)	0.69 (391)	.488

Supplementary Table 60

Differences in vaccination intention regarding the use/non-use of topic-related online information sources – Online messenger (e.g., *WhatsApp, Telegram*)

	Use<br>n=78<br>M (SD)	No use<br>n=315<br>M (SD)	t (df)	p
Vaccination intention	9.12 (3.04)	9.03 (3.39)	0.20 (391)	.842

Supplementary Table 61

Differences in vaccination intention regarding the use/non-use of topic-related online information sources – Online TV & video streaming

	Use<br>n=23<br>M (SD)	No use<br>n=370<br>M (SD)	t (df)	p
Vaccination intention	9.13 (3.28)	9.04 (3.33)	0.12 (391)	.903

Supplementary Table 62

Differences in vaccination intention regarding the use/non-use of topic-related online information sources – Online radio, audio streaming & podcast

	Use<br>n=83<br>M (SD)	No use<br>n=310<br>M (SD)	t (df)	p
Vaccination intention	9.39 (2.97)	8.96 (3.41)	1.04 (391)	.298

Supplementary Table 63

Differences in vaccination intention regarding the use/non-use of topic-related online information sources – Video platforms (e.g., *YouTube*)

	Use<br>n=156<br>M (SD)	No use<br>n=237<br>M (SD)	t (df)	p
Vaccination intention	9.06 (3.33)	9.04 (3.32)	0.08 (391)	.939

Supplementary Table 64

Differences in vaccination intention regarding the use/non-use of topic-related online information sources – Online news sites

	Use<br>n=304<br>M (SD)	No use<br>n=89<br>M (SD)	t (df)	p
Vaccination intention	9.31 (3.12)	8.15 (3.82)	2.64 (124.251)	.009

Supplementary Table 65

Differences in vaccination intention regarding the use/non-use of topic-related online information sources – Medical online consultation

	Use<br>n=4<br>M (SD)	No use<br>n=389<br>M (SD)	t (df)	p
Vaccination intention	6.75 (4.65)	9.07 (3.30)	1.39 (391)	.164

Supplementary Table 66

Differences in vaccination intention regarding the use/non-use of topic-related online information sources – Other online sources

	Use<br>n=13<br>M (SD)	No use<br>n=380<br>M (SD)	t (df)	p
Vaccination intention	9.31 (3.52)	9.04 (3.32)	0.29 (391)	.775
